# A proposal for an additional clinical trial outcome measure assessing preventive effect as delay of events

**DOI:** 10.1007/s10654-012-9752-0

**Published:** 2012-12-07

**Authors:** Per Lytsy, Lars Berglund, Johan Sundström

**Affiliations:** 1 Department of Medical Sciences, Entrance 40, 5th Floor, Uppsala University Hospital, SE-751 85 Uppsala, Sweden; 2 Uppsala Clinical Research Center, Uppsala University Hospital, Uppsala, Sweden

**Keywords:** Clinical trials, Randomized, Kaplan–Meier survival curves, Preventive measures, Treatment Outcome

## Abstract

Many effect measures used in clinical trials are problematic because they are differentially understood by patients and physicians. The emergence of novel methods such as accelerated failure-time models and quantile regression has shifted the focus of effect measurement from probability measures to time-to-event measures. Such modeling techniques are rapidly evolving, but matching non-parametric descriptive measures are lacking. We propose such a measure, the delay of events, demonstrating treatment effect as a gain in event-free time. We believe this measure to be of value for shared clinical decision-making. The rationale behind the measure is given, and it is conceptually explained using the Kaplan–Meier estimate and the quantile regression framework. A formula for calculation of the delay of events is given. Hypothetical and empirical examples are used to demonstrate the measure. The measure is discussed in relation to other measures highlighting the time effects of preventive treatments. There is a need to further investigate the properties of the measure as well as its role in clinical decision-making.

Many chronic diseases develop over long time periods, where the risks of serious adverse symptomatic events increase with time. Prevention aims at reducing such risks, either by reducing the event rate or by delaying the timing of the events. The effect of a preventive intervention is preferably evaluated in a controlled trial where one or more binary outcomes are monitored continuously during the study period. Given such data, there are several ways of examining the effect of the treatment. At any given point in time, the proportions of events in the trial arms may be compared in relative or absolute terms. Other statistical options include the use of time-to-event data to compare the rates, risks or hazards of events during specified time periods. While all these measures are methodologically justified and well used, there is an ongoing debate about which one to prefer, as the choice of effect measure has been shown to affect clinical decision-making [1–7]. The difficulty for physicians and patients to grasp and agree on the chance and magnitude of a preventive treatment evidence based effect is a challenge to informed decision-making, and more generally to the idea of evidence based clinical practice.

This may, however, change with the development of new methods for assessing and illustrating treatment effects, such as accelerated failure-time models (AFT) and quantile regression. AFT models are similar to Cox models, but include a parameterization of the baseline hazard, and give results on the time scale instead of the hazard scale. Quantile regression goes beyond regression models for the conditional mean, and extends the regression model to conditional quantiles of the outcome variable, which offers a more comprehensive analytical approach.[8, 9] Such modeling techniques are rapidly evolving in many scientific fields, including biomedical sciences.[10–12]. In terms of assessing treatment effect, these techniques have shifted the focus from investigating probability measures at specific time points, beyond summary time-to-event measures, to assessment of how the effect develops over time. There has, however, been a lack of a non-parametric descriptive measure that matches these approaches.

In this article we propose an alternative way to illustrate treatment effects from randomized controlled trials, matching the AFT and quantile regression modeling frameworks. By using time-to-event data, it is possible to calculate treatment effect as the delay of events, (DoE) i.e. the time a disease event is delayed due to treatment. We believe that expressing treatment effect as a potential gain in disease-free time is easy to understand for patients, and that the measure, therefore, may be of value in clinical practice.

## Measuring treatment effect as delay of events

**Fig. 1:**
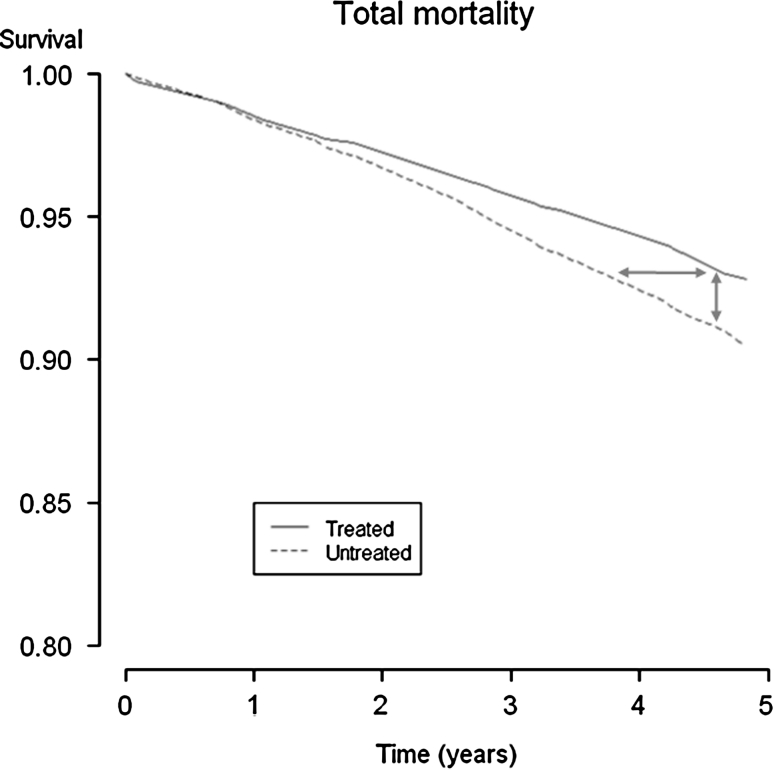
Kaplan–Meier curves for all-cause mortality from the 4S study. The *vertical* difference between the curves represents a difference in proportions, at different time points. The *horizontal* difference between the curves represents a time difference when the cumulative incidence is equal, which corresponds to the time an event is delayed

Assessment of the delay of events may be explained using a Kaplan–Meier graph. The Kaplan–Meier estimator is a non-parametric estimator from incomplete observations, which means that the estimator can account for censored data [13]. This is commonly the case in clinical trials investigating a treatment’s ability to prevent clinically significant adverse events of chronic diseases. Figure 1 presents the Kaplan–Meier curves (survival curves) for the endpoint all-cause mortality in the Scandinavian Simvastatin Survival Study (4S), a randomized controlled trial presenting the first evidence that statin treatment improves survival in patients with coronary heart disease [14]. While the *vertical* difference between the two trial arms represents the difference in proportions of patients still alive at a given point in time, the *horizontal* difference represents a time discrepancy when the study arms have obtained equal proportions or quantiles of survivors. That time difference equals the time delay of the incidence between the groups, in other words the delay of events in patients suffering such events during the study. The delay of events is possible to calculate and plot as a function of follow-up time itself, assuming that the Kaplan–Meier curves are nearly unbiased estimators of the true survival curves [15]. The mathematical expression of the delay of events is explained in appendix.

## Empirical and hypothetical examples

**Fig. 2:**
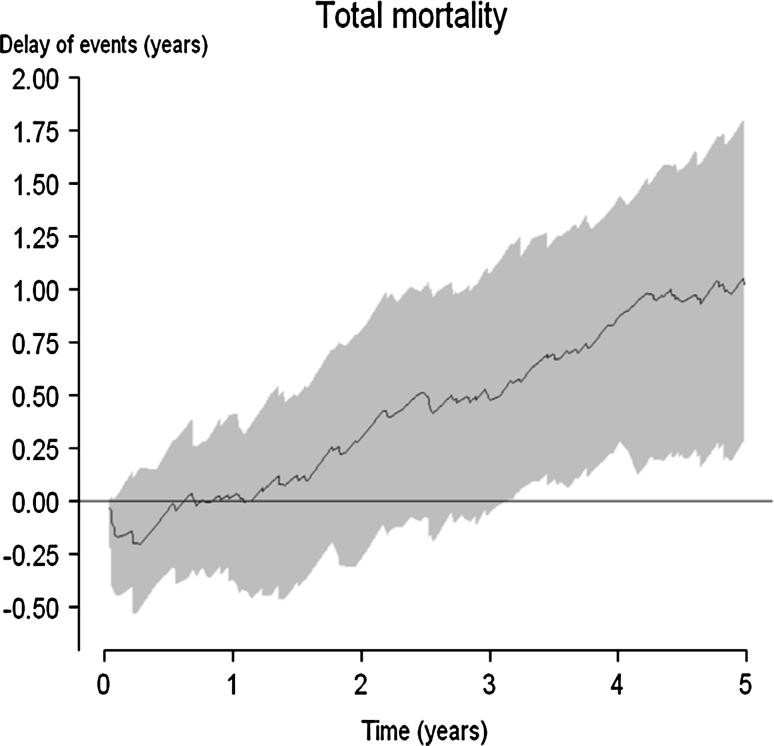
The delay of event curve (with shadowed 95 % confidence intervals) for the endpoint total mortality in the 4S study

Figure 2 presents the delay of events curve (with a shadowed 95 % confidence interval) based on the survival data presented in Fig. 1. The delay of events curve demonstrates no beneficial effect during the first year of treatment. After 3 years of treatment, the delay of events is approximately half a year, and at the end of the study it has reached about 1 year, indicating that persons in the treatment arm who developed an event by the end of the 4S study period had delayed that event for 1 year compared to patients in the control arm.

**Fig. 3:**
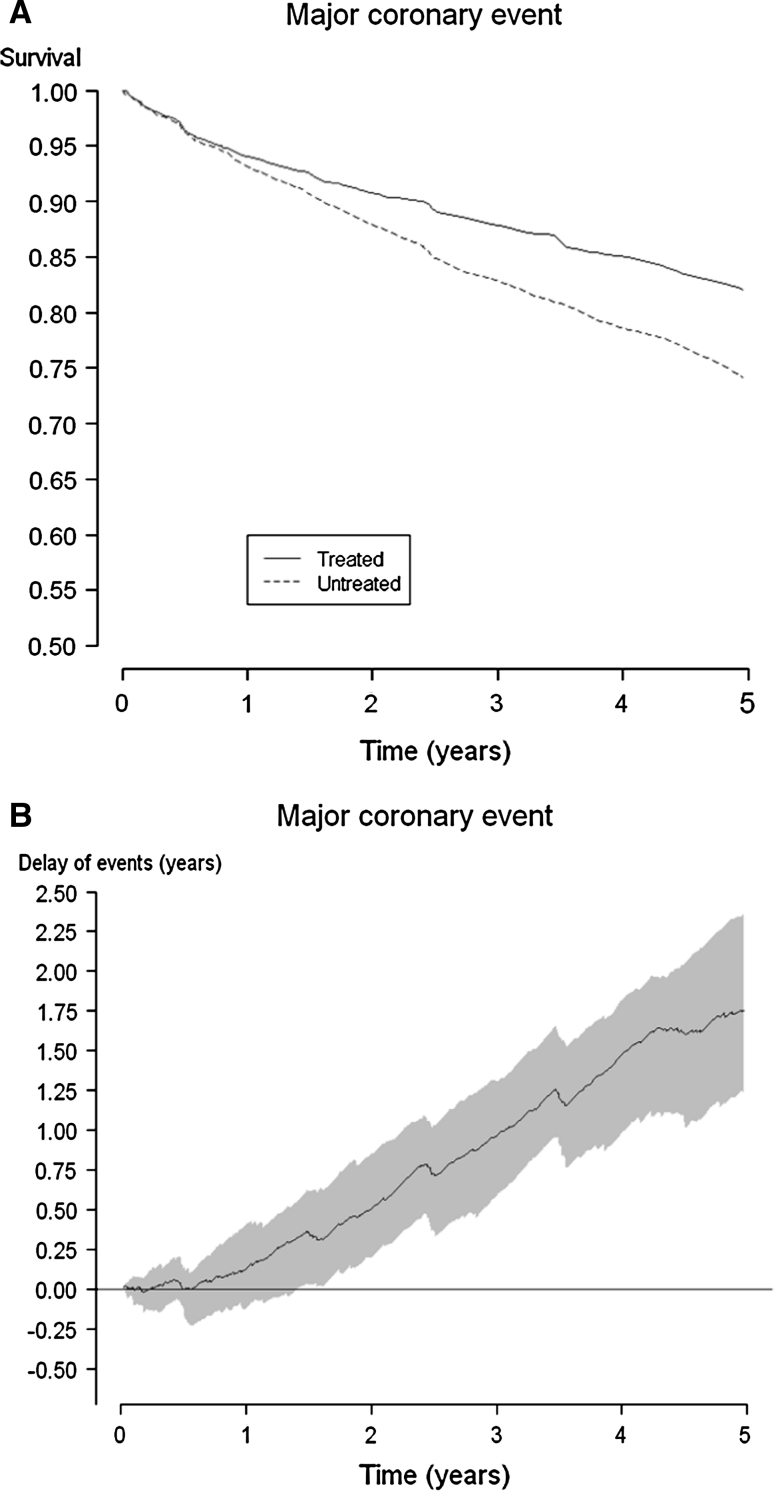
Kaplan-Meier curves for major coronary events* in the 4S study (**a**) and the consecutive delay of event curve with shadowed 95 % confidence interval (**b**). *Major coronary events comprised coronary deaths, definite or probable hospital-verified non-fatal acute MI, resuscitated cardiac arrest, and definite silent MI verified by electrocardiogram

The corresponding Kaplan–Meier and delay of events curves for the endpoint major coronary events in the 4S study are demonstrated in Fig. 3a, b. A statistically significant (at the *P* < 0.05 level) delay of events for endpoint major coronary events is obtained after 1.5 years, and the maximum delay reaches about 1.75 years at the end of the study period.

Generally, the delay of events curve cannot always be expected to increase, not even within a study period. At some point, if the follow-up is long enough, it will decrease until it no longer demonstrates a superior effect, for example due to an aging study sample, competing events, or a time-limited treatment effect. Determining when a delay of events curve falls below a level of effect regarded not to be clinically significant may be of value in order to agree on recommendations for treatment discontinuation.

**Fig. 4:**
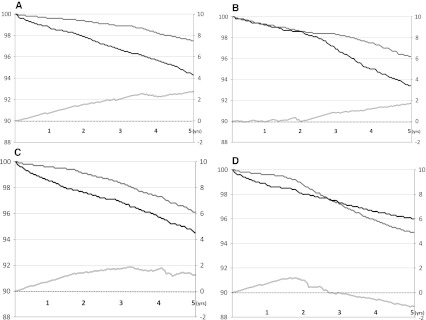
**a** Kaplan Meier curves of four hypothetical intervention studies and their subsequent delay of event curves. *Y-axes* on the *left* correspond to the proportion of event free subjects in the compared groups; *Y-axes* on the *right* represent the time units used in each study. Diverging survival curves will present an increasing delay of events curve within the study time period. **b** Survival curves that are diverging after an initial latency period will present a delay of events curve where the effect is delayed; in this case the effect becomes apparent after about 2 years. **c** Survival curves diverging initially followed by parallel development over time will present a delay of event curve demonstrating a sustained effect, which in this case after 2 years of treatment approximates from between 1 and 2 years until the end of follow-up. **d** Survival curves diverging and crossing over during the study period will demonstrate a delay of events curve where the positive effect seen first diminishes and then provides a negative effect. A negative delay of events curve should be interpreted as if the active treatment causes harm, as demonstrated by a higher event rate in the treated group

Figure 4 presents four hypothetical intervention studies illustrating the delay of events when the survival curves (a) diverge, (b) diverge after an initial latency period, (c) diverge initially followed by parallel survival curves and (d) cross over during the study period.

## Why another effect measure?

It is well known that the established effect measures are associated with some difficulties when used in clinical care for individual decision-making. One problem involves the fact that they are probability measures. Probabilistic thinking is difficult. Laymen, patients, and even skilled professionals all suffer from various degrees of statistical illiteracy, making it difficult for many to perform simple arithmetic calculations and to comprehend risk estimates [16–18]. This predicament is further supported by research showing that the format of the effect measure may influence patients’ acceptance of taking a medication [1, 2, 7] as well as doctors’ and health authorities’ willingness to recommend or prescribe it [3, 19]. This signifies the challenge clinicians face when deciding how to describe treatment outcomes to their patients for the purpose of shared decision-making.

The time-limited follow up in randomized controlled trials might also flaw the understanding of a treatment’s effect, since it does not apply to a patient’s lifetime perspective. The fact that a treatment, relative to a control group, e.g. decreases the risk of death by 30 % may be accurate during the study period, but become less true the longer the results are extrapolated, and is bizarre if extrapolated to a lifetime perspective. For this reason, many health professionals advocate using absolute measures of effect (or its reciprocal: the numbers needed to treat) when presenting treatment effect to patients. However, absolute measures may portray the view that avoidance of events within the study period is the only benefit of a treatment, suggesting that the effect is obtained in a limited number of individuals. There is little support that such an interpretation of beneficial effects from preventive treatment is reasonable, given that no probability measure has the ability to tell if a treatment effect is obtained in a large or a small number of the treated population [20]. It is even possible that every treated patient benefits to a small degree, but that in many patients such advantage will occur beyond the study time frame. Notably, these time constraints also apply to the delay of events, but are more easily spotted here than in probability measures because the delay of events curve (and indeed the Kaplan–Meier curve) highlights variation in treatment effect as a function of time itself.

Based on the understanding of how common diseases, such as cardiovascular disease, develop over a life span, it is likely more correct to assume that prevention postpones disease events rather than entirely avoids them. From the perspective of individuals, it would therefore be of value to report the time an event may be delayed, rather than a probability measurement of the likelihood of being event-free at a given time point. That kind of reasoning is well adopted in other medical fields, such as oncology, in which randomized controlled trials often continue until a defined proportion of patients in the study groups have developed a certain endpoint. The effect of treatment in such studies is thus reported as a gain in disease-free time. Another medical field emphasizing time as a major dimension of interest is global health, in which life expectancy and quality-adjusted life years frequently are used as measures of health and disease burden. Further, the delay of events is a descriptive measure that conceptually matches the increasingly recognized AFT and quantile regression modeling techniques.

## Clinical use of the delay of events

It has been shown that presenting effect as gain in event-free time, rather than cumulative probability, seems to increase a treatment’s attractiveness [21]. Furthermore, the size of the time delay seems to be related to peoples’ motivation to take a medication [22].

If a patient is asked to presume that he or she will develop the event within the length of the study period, the delay of events will serve as an estimation of the magnitude of the treatment effect developing over time. Based on the delay of events curve from the 4S study, patients eligible for the treatment used in that study might be told the following: “You have an unnecessarily high risk of developing a major coronary event. No one can tell for sure if or when this will occur in your case. Presuppose that you actually would develop this event within the next 5 years; then taking this treatment during that time will postpone the event by up to approximately 1.75 years.” Hence, the delay of events curve from a trial will serve as an estimate of relevance for most individuals eligible for treatment.

## Critical appraisal of the delay of events

The delay of events curve is an alternative way to summarize and describe time-to-event data, and as such the curve will exhibit the same properties and restraints as Kaplan–Meier curves. Calculating the delay of events curve does not require any assumptions to be made about the distribution of the data.

There are several other measures of effect highlighting the time perspective. There are models that estimate the mean residual life and cumulative treatment effects [23, 24] as well as direct assessments of the gain in life expectancy [25, 26]. The gain in life expectancy compares mean (event-free) survival times in two study groups, and hence demands a follow-up until every patient and control has died (or developed the event). Another way to assess a treatment’s effect as a time variable is the gain in median survival time. The median survival time measure demands a follow-up until at least half of the study groups have died or developed the event, and is thus rarely convenient as an outcome measure in studies assessing rare events, which is commonly the case in preventive medicine. The delay of events curve has an advantage in that sense, since it is also possible to calculate in studies with low event rates and high numbers of right-censored patients.

Most measures utilizing survival time in clinical trials are variants of the relation between the areas under event-free curves at a given time point, which are two-dimensional measures of person-years. These areas reflect the entire event occurrences in the study arms during follow-up until that time point, and are hence summary measures. Their relation cannot be used to calculate a difference in time to attain a certain cumulative incidence, as it includes events when the worse-off group has reached a cumulative incidence that is not reached by the better-off group.

Conceptually, the delay of events applies best to outcomes that are inevitable, such as mortality. If the delay of events is assessed for other outcomes, it is important to regard and manage the possibility of competing risks, where one option might be using composite endpoints of the event of interest and death from other cause. It is suggested that the problem with competing risks is an area for future research for the measure.

In theory, presenting an effect as delay of events is most appropriate when assessing the effect of prevention of chronic disease events. The method may, however, be used for any intervention influencing the timing of adverse clinical events.

As this is a new effect measure, several questions remain to be answered. These include determination of the influence of potential confounders on the outcome; how the accuracy of the results is affected by the sample size, and how the measure relates to subgroups of patients with different baseline risks. There is also a need to discuss and establish guidelines about how the measure should be used, presented and interpreted within specific research areas. Such guidelines might include directives of a priori defined time points, or quantiles of survival-time of interest, as well as determining what effect should be regarded as clinically significant at these time points or quantiles. It is also suggested that future research investigate the measure’s potential value and limitations when using observational data, such as cohort studies.

When Wright and Weinstein standardized gains in life expectancy from a variety of medical interventions, they concluded that a life gain of 1 month or more following a preventive intervention was to be considered large in populations with average risk [26]. What patients regard as significant in terms of delay of disease probably depends on several factors, including their individual situations, knowledge of the disease and the therapy (including awareness of side-effects) as well as their attitudes and intrinsic values. Thus, there is a need to investigate how treatment effect expressed as delay of events is valued in different populations, and how it affects decision-making.

## Conclusion

The delay of events is an effect measure that may be calculated using time-to-event data. The measure describes preventive treatment effect as the time an event may be delayed due to treatment. We believe this way of presenting treatment effect is easy to understand for individuals, making it suitable for use in the clinical situation when physicians explain outcomes to patients. The delay of events measure should not replace the established efficacy measurements. Rather, it is suggested that it be considered a complementary way to present treatment effect from clinical trials. Since this is a new effect measure, there is a need to further understand its strengths and limitations, as well as investigate how it affects clinical decision-making.

## Appendix

Consider right-censored data with $n_{i} (i \, = { 1, 2) }$ observations from two independent groups of individuals, where Group 1 is the better-off group and Group 2 is the worse-off group:

$$ (t_{11} , d_{11} ), (t_{12} , d_{12} ), \ldots , (t_{{1n_{1} }} , d_{{1n_{1} }} )\quad {\text{and}}\quad  (t_{21} , d_{21} ), (t_{22} , d_{22} ), \ldots , (t_{{2n_{2} }} , d_{{2n_{2} }} ) { } $$

where $t_{ij}$ is the survival or censoring time for the *j*th observation from group *i,*

$$ d_{ij} = 1 \quad {\text{ if }}\, t_{ij} {\text{ is uncensored and }}\quad d_{ij} = 0 \quad {\text{ if }}t_{ij} {\text{ is censored}} . $$

For convenience we assume that $t_{i1} < t_{i2} < t_{i3} < \cdots < t_{{in_{i} }} .$

The observed Kaplan–Meier curves $s_{i} (t)$ are given by the formula

$$ s_{i} (t) = \coprod\limits_{j = 1}^{{k_{it} }} {\left( {\frac{{n_{i} - j}}{{n_{i} - j + 1}}} \right)}^{{d_{ij} }} . $$

Here *k*_*it*_ is the value of *k*_*i*_ such that $t_{i} \in [t_{{k_{i} }} , t_{{k_{i} + 1}} ].$

The aim is to estimate the difference *D*(*t*) (delay of event) in time when the groups show

equal survival incidence, expressed as a function of time. The estimator of *D(t)* is *d(t)* which is given by ÷

$$ d(t) = s_{1}^{ - 1} (s_{2} (t)) - t. $$

A confidence interval for *D*(*t*) may be obtained with the bootstrap percentile method: 10,000 bootstrap samples of (*t*_*ij*_ *; d*_*ij*_) are drawn and from each sample and *d(t)* is calculated in each bootstrap sample. The 2.5 and 97.5 percentiles in the distribution of the 10,000 estimates are the limits of a 95 % confidence interval for *D*(*t*).
